# Contribution of community-based newborn health promotion to reducing inequities in healthy newborn care practices and knowledge: evidence of improvement from a three-district pilot program in Malawi

**DOI:** 10.1186/1471-2458-13-1052

**Published:** 2013-11-07

**Authors:** Jennifer A Callaghan-Koru, Bareng AS Nonyane, Tanya Guenther, Deborah Sitrin, Reuben Ligowe, Emmanuel Chimbalanga, Evelyn Zimba, Fannie Kachale, Rashed Shah, Abdullah H Baqui

**Affiliations:** 1 International Center for Maternal and Newborn Health, Johns Hopkins Bloomberg School of Public Health, Baltimore, MD, USA; 2 Save the Children, Washington, DC, USA; 3 Save the Children, Lilongwe, Malawi; 4 Department of Reproductive Health, Malawi Ministry of Health, Lilongwe, Malawi

**Keywords:** Newborn health, Community health workers, Equity, Malawi, Health surveillance assistants, Maternal health

## Abstract

**Background:**

Inequities in both health status and coverage of health services are considered important barriers to achieving Millennium Development Goal 4. Community-based health promotion is a strategy that is believed to reduce inequities in rural low-income settings. This paper examines the contributions of community-based programming to improving the equity of newborn health in three districts in Malawi.

**Methods:**

This study is a before-and-after evaluation of Malawi’s Community-Based Maternal and Newborn Care (CBMNC) program, a package of facility and community-based interventions to improve newborn health. Health Surveillance Assistants (HSAs) within the catchment area of 14 health facilities were trained to make pregnancy and postnatal home visits to promote healthy behaviors and assess women and newborns for danger signs requiring referral to a facility. “Core groups” of community volunteers were also trained to raise awareness about recommended newborn care practices. Baseline and endline household surveys measured the coverage of the intervention and targeted health behaviors for this before-and-after evaluation. Wealth indices were constructed using household asset data and concentration indices were compared between baseline and endline for each indicator.

**Results:**

The HSAs trained in the intervention reached 36.7% of women with a pregnancy home visit and 10.9% of women with a postnatal home visit within three days of delivery. Coverage of the intervention was slightly inequitable, with richer households more likely to receive one or two pregnancy home visits (concentration indices (CI) of 0.0786 and 0.0960), but not significantly more likely to receive a postnatal visit or know of a core group. Despite modest coverage levels for the intervention, health equity improved significantly over the study period for several indicators. Greater improvements in inequities were observed for knowledge indicators than for coverage of routine health services. At endline, a greater proportion of women from the poorest quintile knew three or more danger signs for pregnancy, delivery, and postpartum mothers than did women from the least poor quintile (change in CI: -0.1704, -0.2464, and -0.4166, respectively; p < 0.05). Equity also significantly improved for coverage of some health behaviors, including delivery at a health facility (change in CI: -0.0591), breastfeeding within the first hour (-0.0379), and delayed bathing (-0.0405).

**Conclusions:**

Although these results indicate promising improvements for newborn health in Malawi, the extent to which the CBMNC program contributed to these improvements in coverage and equity are not known. The strategies through which community-based programs are implemented likely play an important role in their ability to improve equity, and further research and program monitoring are needed to ensure that the poorest households are reached by community-based health programs.

## Background

There is strong and growing evidence of inequities in maternal and newborn health worldwide. The term “inequity in health” describes unjust differences in access to care and health outcomes between advantaged and disadvantaged population groups [1]. At the global level, the populations of wealthier countries have better maternal and neonatal health outcomes than poorer countries [2,3]. Sub-Saharan Africa, the poorest region in the world, accounts for 14 of the 18 countries with the highest neonatal mortality rates globally [2]. Socioeconomic inequality is also consistently observed within many countries. In both rich and poor countries, more neonatal deaths occur among the poor [2] and rural areas have higher mortality rates, missing out on the great declines in mortality that occurred in urban areas during the 1990s [4]. It is estimated that elimination of within-country inequities in high mortality countries could reduce annual neonatal deaths by close to 750,000 [2].

Several factors may contribute to wealth-based inequities in child and newborn health outcomes, including living conditions, knowledge and behaviors, nutritional status, and physical and financial access to health services [5-9]. Although evidence-based interventions exist to address the main causes of newborn death [10], there is inadequate coverage of these interventions among the poor (a trend sometimes referred to as the “inverse care law” [11]). For example, while virtually all deliveries in high-income countries have a skilled attendant [12], 54% is the median coverage for skilled attendance at birth among the 68 low-income countries tracked by the Countdown to 2015 [13]. Within poor countries, richer women are between 2 and 3 times more likely to utilize antenatal care and up to six times more likely to have a skilled attendant at delivery [13-15]. In some countries these equity gaps have remained consistent since 1990 [16]. There is consensus that reducing inequities is critical for making major gains in newborn and child health and reaching Millennium Development Goal 4 [2].

Although there are multiple approaches that can be used in pursuit of greater equity in health, community-based programs and outreach are considered especially important [15,17,18]. Community-based programs may quickly expand coverage to areas where access to facility care is limited, thereby removing key barriers for poor households such as distance and transport costs. Community-based programs are also often offered free of charge and can be used to promote healthy behaviors, to provide screening and referral for complications and illnesses, to promote utilization of facility-based services, and in some cases to provide treatment at the home or community level.

Over the past decade, Saving Newborn Lives (SNL) of Save the Children helped develop evidence that community-based programs are effective at reducing neonatal mortality through support of trials in South Asia [19-21] and ongoing trials in Africa [22-24]. SNL has also successfully advocated for policy changes to expand community-based newborn programs in many countries [25,26]. However, little is currently known about the extent to which the community delivery strategy improved equity in newborn health.

This paper examines the contributions of community-based programming towards improving the equity of newborn health in Malawi, one of the countries included in SNL’s initiative to scale-up community-based newborn care programs in partnership with governments. Although large inequities have been reported in Malawi for utilization of maternal and child health services [27], little is known about inequities in knowledge and practices related to newborn care. The objectives of this paper are to: 1) describe baseline coverage of maternal and newborn health services, knowledge, and newborn care practices by household wealth status; 2) to assess changes in health inequities following implementation of a community-based program for promotion of newborn health.

## Methods

### Study setting

A land-locked East African country of over 13 million people [28], Malawi is ranked among the ten poorest countries in the world. The Gross National Income per capita is $290 [29] and development assistance accounts for a large part of the Malawi’s economy at $49 per capita [30]. The great majority of Malawi’s population can be considered poor by global standards, with over 90 percent of the population living on less than $2 per day [29]. Because of the overall poverty, general inequality measures for Malawi are not high when compared with many of Malawi’s richer neighbors [30]. However, there is evidence of inequities in several maternal and child health indicators, including skilled attendance at delivery (poorest: 65%; least poor: 90%), postnatal care for women (poorest: 38%; least poor: 52%), child mortality, and care seeking and nutritional status for children under age five [27].

The community-based maternal and newborn care pilot program was implemented in selected areas of three rural districts in Malawi—Thyolo, Dowa, and Chitipa. The major occupation in rural areas of Malawi is farming, and 64% of the population is considered literate [28]. Although the area is rural, the population is dense with an average of 139 persons per square kilometer [28]. Nationally, the neonatal mortality rate for Malawi is estimated at 31 deaths per 1,000 live births and under-five mortality is 112 per 1,000 [27]. A key challenge to improving maternal and newborn health in Malawi is access to quality health services. It is estimated that only 54% of the population resides within a 5-km radius of a health facility [31]. Patients who reach health facilities encounter a critical shortage of health staff in Malawi, where there are approximately 7 doctors and 37 nurses per 100,000 population [32], more than 20 times fewer than in the United States [33].

The expansion of Malawi’s existing cadre of community-based health workers, known as Health Surveillance Assistants (HSAs), is considered an important strategy for improving access to primary care for rural populations [31,34]. HSAs serve rural communities with a target catchment area of 1,000 population, although many HSA catchment populations exceed this target and can reach more than 2,500 population. MOH policy is that HSAs should be recruited from within the district where they will work, but many have been recruited centrally and are not from the communities where they are posted. HSAs’ main responsibility is to provide health education and hygiene promotion, and they receive 10 to 12 weeks of training for this role. In addition, they often receive in-service training for various vertical program activities, including community case management of childhood illness, family planning, tuberculosis drug distribution, water and sanitation, immunization and growth monitoring for children under age 5, providing community therapeutic feeding, voluntary counseling and testing for HIV/AIDS, and following up patients on antiretroviral therapy at community level [35]. Prior to this intervention, HSAs were not trained to provide home visits or counseling on newborn care.

### Study design and intervention

This study is a before-and-after evaluation of Malawi’s Community-Based Maternal and Newborn Care (CBMNC) program, a package of facility and community-based interventions to improve newborn health implemented by the Malawi Ministry of Health (MOH) with support from Save the Children’s SNL program and UNICEF. Three districts—Thyolo, Dowa, and Chitipa—were chosen for the pilot study in collaboration with the MOH. These districts represent Malawi’s three geographic regions (north, central, and south), and were also selected based on overall representation in relation to child and neonatal mortality indicators, progress in implementation of the Accelerated Child Survival and Development and Integrated Management of Childhood Illness programs, as well as district interest in participation. Seven health facilities in Dowa, 7 in Chitipa, and 8 facilities in Thyolo, were selected for the intervention, with a total catchment population of approximately 711,000. The selection criteria for participating health facilities within each district included the size of the facility’s catchment area (with a preference for larger catchment populations), the presence of at least two health workers on staff, and the interest of the facility staff in participation in the pilot. Among the 24 intervention facilities, eight were district or rural community hospitals and 14 were health centers. A decision was made not to include comparison areas in the study due to a simultaneous rapid scale-up of the same CBMNC package by the MOH and multiple partners across the three districts.

At the facility level, health workers received a 21-day in-service training on integrated maternal and newborn care (IMNC), which included modules on newborn resuscitation, basic newborn care, (thermal protection, hygienic cord care, breastfeeding support, etc.), kangaroo mother care for low birth weight babies, and identification and referral of newborns with signs of infection. Ninety-six facility staff completed training on IMNC in the pilot areas between July and September 2009.

At the community level, a total of 622 HSAs in the pilot areas received a 10-day training in Community Based Maternal and Newborn Care (CBMNC) with modules on care during pregnancy and delivery, immediate newborn care, postnatal care for mother and newborn, breastfeeding, identification of newborn danger signs, management of low birth weight babies and conducting home visits. Following training, HSAs were instructed to create a register of women of childbearing age in their catchment areas and update the list every two months through home visits and discussions with community leaders to identify current pregnancies. After identifying pregnant women, HSAs were expected to make 3 home visits during pregnancy (one per trimester) and 3 postnatal home visits for mothers and newborns (on days 1, 3 and 8 for all births). During home visits, HSAs promoted a package of health behaviors (see Table 1) and assessed women and newborns for danger signs requiring referral to a health facility. Seventy-five percent of the HSAs received additional community mobilization training to establish “core groups” of community members that would conduct health education, generate demand for services, and inform HSAs of pregnancies and deliveries. Trainings of HSAs in pilot areas started in July 2008 and were completed by October 2010*.* The Ministry of Health, Save the Children, and UNICEF conducted quarterly supervision of the CBMNC package to reinforce training and implementation. HSAs were selected for supervision visits based on assessments of their performance and reporting. During visits to HSAs’ communities, supervisors mentored HSAs in counseling during home visits and correctly completing registers. The District Executive Committees and District Development Committees were oriented to the program by the District Health Office, while HSAs were responsible for sensitizing the communities in which they worked to the new service through home visits and their meetings with Village Health Committees.

**Table 1:** Content of prenatal and postnatal home visits by HSAs

**Pregnant women**		
**Visit 1 (1**^**st **^**trimester, if possible)**	**Visit 2 (2**^**nd **^**trimester)**	**Visit 3 (3**^**rd **^**trimester)**
Counseling on:	Counseling on:	Counseling on:
• Early ANC including IPTp, ITN, TTV	• Early recognition of danger signs and prompt care seeking among pregnant women	• Skilled attendance at birth
• Minor ailments of pregnancy & management/care seeking	• Birth preparedness and complication readiness	• Clean delivery/Clean delivery kit
• Good nutrition	• Subsequent visits for ANC including IPTp, ITN, TT	• Early initiation and exclusive breastfeeding
• Hygiene and rest	• Prevention of mother-to-child HIV transmission (PMTCT)	• Newborn warmth, asphyxia management, skin-to-skin, delaying first bath
• Danger signs of pregnancy		• PMTCT and feeding options
• Ascertaining HIV status		• Family planning
		• Common newborn and maternal danger signs
**Postnatal visits**		
**Day 1 visit**	**Day 3 visit**	**Day 7 visit**
**(Home delivery)**	**(Both facility and home delivery)**	**(Both facility and home delivery)**
Counseling on newborn care:	Counseling on newborn care:	Counseling on newborn care:
• Attachment and positioning	• Attachment and positioning	• Attachment and positioning
• Early initiation and exclusive breastfeeding	• Exclusive breastfeeding	• Exclusive breastfeeding
• Warmth/skin to skin/delay first bath	• Vaccinations	• Vaccinations
• Hygiene, cord care/skin care	• Hygiene, cord care and skin care	• Hygiene, cord care and skin care
• Support PMTCT when necessary	• Warmth, skin-to-skin care	• Warmth, skin-to-skin care
Activities:	Activities:	Activities:
• Examine newborn & identify: danger signs or low birth weight babies & refer	• Examine newborn & identify: danger signs or low birth weight babies & refer	• Examine newborn & identify: danger signs or low birth weight babies & refer
	• Encourage postnatal check at a health facility on day 7	• Subsequent weekly visits for low birth weight babies
Counseling on maternal care:	Counseling on maternal care:	Counseling on maternal care:
• Identify maternal danger signs and refer	• Identify maternal danger signs and refer	• Identify maternal danger signs and refer
• Good nutrition	• Good nutrition	• Good nutrition
• Hygiene	• Hygiene	• Hygiene
• Rest	• Rest	• Rest
		• Encourage under-five visits and family planning at 6 weeks

### Data collection

A household survey was conducted at baseline and endline to measure the coverage of facility-based maternal and newborn care services, home visits by HSAs (endline only), knowledge about maternal and newborn danger signs, and newborn care practices. The baseline survey took place prior to the start of intervention activities in November-December 2007, and the endline survey was conducted in May-June 2011. Each survey included a random sample of 900 women, allowing us to detect, for the majority of indicators, a difference of at least 10 percentage points between baseline and endline with the pooled sample across districts and 20 percentage points within each district, accounting for a design effect of 2 with 80% power [36].

Households were selected for the survey following a two-stage cluster sampling design adapted from the Expanded Program on Immunization (EPI) survey methodology [37]. In the first stage, 90 enumeration areas, 30 per district, were randomly sampled with probability proportional to size. Within each cluster, teams interviewed 10 households with at least one woman 15–49 years of age who had a live birth in the last 12 months. Households were selected following the EPI random walk method, whereby teams randomly selected a starting household in the center of the cluster, and after choosing a random direction, proceeded to each adjacent household until identifying an eligible woman [38]. In the case that a selected household had more than one eligible woman, one woman was randomly selected to complete the interview.

The sampling frame for clusters in the baseline survey was composed of all census enumeration areas within the three districts. For the endline survey, the sampling frame was restricted to only the census enumeration areas corresponding with the catchment areas of the 458 HSAs who had been trained by December 2009, to allow at least 15 months of program implementation. In both baseline and endline surveys, the sample was evenly divided between districts with 30 clusters per district.

Data collection was conducted by 12 experienced interviewers who had either completed a university education or were university students. Data collectors were trained for 3 days and data collection took 13 days for both baseline and endline surveys. Each data collection team had a supervisor responsible for checking questionnaires for correctness and completeness and overseeing sampling and other survey procedures in the field. A full-time survey coordinator provided supervision and quality assurance for the overall implementation of the survey and SNL staff provided additional oversight and monitoring throughout the survey process. At baseline, data were entered into MS Access and at endline data were double entered into SPSS and discrepancies were reconciled with reference to the original survey form.

### Analysis

#### Measurement of knowledge, coverage and practices for newborn health

All variables on coverage, knowledge, and practices were measured dichotomously. Postnatal home visits by HSAs were considered to be within three days if reported to have occurred within 72 hours after birth or on day 0, 1, 2, or 3. Skilled providers for antenatal care included doctors, clinical officers, nurses and midwives. Immediate breastfeeding was defined as initiation ≤1 hour after birth. Delayed bathing was defined as the newborn’s first bath given ≥6 hours after birth. Skin-to-skin contact was based on whether the mother reported the baby was placed in skin-to-skin contact with her 'as soon as s/he was born’. Records that were missing information for a specific indicator were excluded from the calculation of that indicator. Full indicator definitions are provided in Additional file 1: Table S1.

#### Assessment of socioeconomic status and equity

In order to assign households to wealth quartiles, an asset score was generated using principal components analysis. All assets included in the index are presented in Table 2. The first principal component accounted for 18% of variation at baseline and 17% of variation at endline. Indicators were calculated by wealth quintile separately for baseline and endline with robust standard errors adjusting for clustering [39].

**Table 2:** Background characteristics and assets included in the sample

	**Baseline**	**Endline**
	**n (%)**	**n (%)**
**Respondent characteristics**		
***Age***		
15 to 19	115 (13%)	131 (15%)
20 to 29	474 (53%)	507 (56%)
30 to 39	208 (23%)	215 (24%)
40 to 49	29 (3%)	24 (3%)
Don’t know	74 (8%)	23 (3%)
***Schooling***		
No school	129 (14%)	111 (12%)
Primary school	635 (70%)	631 (70%)
Secondary school	135 (15%)	154 (17%)
Higher than secondary	2 (0.2%)	2 (0.2%)
***Toilet facilities***		
Flush toilet	10 (1%)	9 (1%)
Improved pit latrine	163 (18%)	131 (15%)
Unimproved pit latrine	680 (75%)	739 (82%)
No toilet/bush facilities	41 (5%)	21 (2%)
***Electricity and appliances***		
Electricity	31 (3%)	26 (3%)
Radio	629 (70%)	530 (59%)
Television	28 (3%)	53 (6%)
Mobile phone	131 (15%)	310 (34%)
Nonmobile phone	14 (2%)	15 (2%)
Refrigerator	6 (1%)	12 (1%)
***Cooking fuel***		
Electric or gas	0	9 (1%)
Coal	44 (5%)	27 (3%)
Wood	855 (95%)	864 (96%)
***Roof material***		
Iron sheets or tiles	345 (38%)	238 (26%)
Grass or leaves	554 (61%)	661 (73%)
***Wall material***		
Fired bricks	434 (48%)	396 (44%)
Unfired bricks	360 (40%)	261 (29%)
Stone	0	8 (1%)
Dirt of grass	99 (11%)	233 (26%)
***Household items***		
Watch	413 (46%)	265 (29%)
Bicycle	474 (53%)	434 (48%)
Motorcycle	46 (5%)	19 (2%)
Animal-drawn cart	21 (2%)	30 (3%)
Car	2 (0.2%)	5 (0.6%)
***Livestock***		
Cattle	172 (19%)	92 (10%)
Donkeys	3 (0.3%)	4 (0.4%)
Sheep	47 (5%)	5 (0.6%)

We calculated concentration indices for each indicator of interest as a single measure of inequality. The concentration index (CI) measures the area between the concentration curve and the line of perfect equality [40]. CI values range between -1 and +1; a positive value indicates inequality favoring the rich, a negative value indicates inequality favoring the poor, and a CI closer to zero indicates near perfect equality. Concentration indices have been increasingly used to assess inequalities in maternal and child health outcomes [41,42]. We generated concentration curves from the wealth scores using the generalized Lorenz curve approach. From these curves we derived the CIs using regression models with robust standard errors to account for clustering [42]. The change in CI between baseline and endline for each indicator was calculated and then tested using the t-test. All analyses were conducted in Stata 11 [43].

### Ethical considerations

Oral informed consent was obtained from all survey respondents. Ethical review and approval was provided by the Malawi National Health Sciences Research Committee (protocol number 473).

## Results

The baseline household survey sample included 903 women, the majority of whom were between the ages of 20 and 39 (Table 2). Seventy percent of women had some primary school education, while 14% reported no education and 15% reported some secondary or post-secondary education. The majority of households had an unimproved pit latrine (75%), cooked with wood fuel (95%), lived in homes with a natural roof of grass or leaves (61%), and had walls made of bricks (88%). Few households possessed electronics or appliances other than a radio (70%), although watch and bicycle ownership was reported by around half of households. The endline household survey included a sample of 900 women. Although the sampling frame was different between the two surveys, the samples are comparable in terms of demographic indicators and most household assets. Slightly more households in the endline household survey reported inferior home construction materials, such as dirt walls (15 percentage points higher) and a grass or leaf roof (12 points higher), and fewer households reported watch or bicycle ownership. Notably, mobile phone ownership increased from 15% at baseline in 2007 to 34% at endline in 2011. The increase in mobile phone ownership may also explain some of the difference in ownership of a wristwatch between surveys, as the mobile phone may replace the wristwatch as a time keeping device.

Table 3 presents the coverage and equity achieved by the community-based intervention as measured in the endline survey. Thirty-six percent of women received at least one pregnancy home visit by an HSA, while only 10.9% received a post-delivery home visit within the recommended 3-day window. Coverage of one and two pregnancy home visits was significantly higher among women in the least poor quartiles, as evidenced by the positive concentration indices of 0.0788 and 0.0960, respectively. Postnatal home visits were not significantly higher among less poor households. Thirty-four percent of women reported being aware of a core group in their community, and although core groups were not expected to make regular home visits to pregnant women, 9.6% received a home visit from a core group member during pregnancy. Awareness of and contact with a core group member were not significantly inequitable.

**Table 3:** Coverage of community-based intervention activities, by quartile

	**Endline**
**At least one home visit by HSA during pregnancy**	
Overall	36.7
Poorest quartile	29.0
2nd quartile	37.2
3rd quartile	37.7
Least poor quartile	42.8
	0.0786
Concentration index (95% Confidence interval)	(0.0307, 0.1265)
**Two or more home visits by HSA during pregnancy**	
Overall	21.2
Poorest quartile	16.3
2nd quartile	20.9
3rd quartile	21.3
Least poor quartile	26.2
	0.0960
Concentration index (95% Confidence interval)	(0.0219, 0.1701)
**At least one postnatal home visit by HSA within 3 days**	
Overall	10.9
Poorest quartile	7.8
2nd quartile	12.1
3rd quartile	12.0
Least poor quartile	11.9
	0.0933
Concentration index (95% Confidence interval)	(-0.0237, 0.2104)
**Two or more postnatal home visits by HSA**	
Overall	4.7
Poorest quartile	2.7
2nd quartile	8.4
3rd quartile	4.9
Least poor quartile	2.7
	-0.0399
Concentration index (95% Confidence interval)	(-0.1530, 0.0733)
**Aware of a “core group” present in community**	
Overall	34.1
Poorest quartile	33.8
2nd quartile	31.4
3rd quartile	35.3
Least poor quartile	36.0
	0.0241
Concentration index (95% Confidence interval)	(-0.0411, 0.0894)
**Received visit from core group member during pregnancy**	
Overall	9.6
Poorest quartile	6.2
2nd quartile	10.2
3rd quartile	11.6
Least poor quartile	10.2
	0.1015
Concentration index (95% Confidence interval)	(-0.0090, 0.2119)

The knowledge of maternal and newborn danger signs greatly increased between the baseline and endline survey (Table 4). Knowledge of three or more postpartum danger signs increased by a factor of 6, from 5.5% at baseline to 35.0% at endline. Knowledge of newborn danger signs achieved the highest coverage in the endline survey, with 60.1% of respondents able to list 3 or more newborn danger signs, compared to 13.4% at baseline. The concentration indices for three of the four knowledge indicators changed from positive (pro-rich) to negative (pro-poor) between baseline and endline, although the endline CIs are not statistically significantly pro-poor. The change in CI for all four knowledge indicators is negative, indicating improvements in equity, and statistically significant for pregnancy, delivery, and postpartum danger signs. Relatively large improvements in the knowledge gap between poor and less poor households were observed for delivery danger signs (CI: -0.2464) and postpartum danger signs (CI: -0.4166).

**Table 4:** Knowledge of danger signs for maternal and newborn health, by wealth quartile

	**Baseline**	**Endline**	**Change in CI**
**Knows 3 or more pregnancy danger signs (12 signs total)**			
Overall	8.1	43.9	
Poorest quartile	4.3	46.7	
2nd quartile	8.1	48.2	
3rd quartile	11.9	41.1	
Least poor quartile	8.1	39.6	
	0.1378	-0.0327	-0.1704
Concentration index (95% confidence interval)	(0.0207, 0.2548)	(-0.0784, 0.0131)	(-0.2945, -0.0463)
**Knows 3 or more delivery danger signs (6 signs total)**			
Overall	5.2	17.2	
Poorest quartile	4.3	18.2	
2nd quartile	3.6	12.1	
3rd quartile	3.1	19.6	
Least poor quartile	9.9	12.9	
	0.2234	-0.0229	-0.2464
Concentration index (95% confidence interval)	(0.0123, 0.4346)	(-0.1140, 0.0681)	(-0.4733, -0.0194)
**Knows 3 or more postpartum danger signs (10 signs total)**			
Overall	5.5	35.0	
Poorest quartile	3.4	34.7	
2nd quartile	1.8	38.9	
3rd quartile	5.8	34.8	
Least poor quartile	11.2	31.6	
	0.4086	-0.0079	-0.4166
Concentration index (95% confidence interval)	(0.1245, 0.6928)	(-0.0726, 0.0567)	(-0.7044, -0.1288)
**Knows 3 or more newborn danger signs (12 signs total)**			
Overall	13.4	60.1	
Poorest quartile	10.7	55.1	
2nd quartile	12.7	62.8	
3rd quartile	15.0	58.0	
Least poor quartile	15.2	64.4	
	0.1271	0.0328	-0.09423
Concentration index (95% confidence interval)	(-0.0048, 0.2589)	(-0.0012, 0.0669)	(-0.2289, 0.0402)

Table 5 presents the coverage of facility-based MNH services and newborn care behaviors at baseline and endline. The overall coverage for all indicators collected at baseline increased between the two surveys, with the greatest increases observed for facility delivery (21 percentage points) and breastfeeding within the first hour (19.8 percentage points). The smallest increase was observed for attendance at antenatal care, which was high at baseline, at 89.3% percent. The CIs for all indicators at baseline and endline are relatively small and close to zero, indicating that no strong inequities were observed. At baseline, the concentration indices for all indicators either significantly favored the least poor, or were not significant in either direction. At endline, only two indicators had statistically significant pro-rich inequities: facility delivery (CI: 0.0131), and skin-to-skin contact (CI: 0.0492), which was not measured at baseline. In contrast, the endline CI for delayed bathing (-0.0252) significantly favored poorer households. The differences in CIs showed small, yet statistically significant, decreases in inequities between baseline and endline for facility delivery (-0.0591), early breastfeeding (-0.0379) and delayed bathing (-0.0405).

**Table 5:** Coverage of health behaviors, by wealth quartile

	**Baseline**	**Endline**	**Change in CI**
***Maternal and newborn care at facility***			
**At least 1 ANC visit with a skilled provider**			
Overall	89.3	94.8	
Poorest quartile	83.3	93.3	
2nd quartile	91.9	92.9	
3rd quartile	85.8	96.9	
Least poor quartile	96.5	96.0	
	0.0258	0.0058	-0.0200
Concentration index (95% confidence interval)	(0.0068, 0.0448)	(-0.0057, 0.0173)	(-0.0420, 0.0019)
**Delivery at a health facility**			
Overall	70.6	91.6	
Poorest quartile	57.2	88.4	
2nd quartile	71.0	90.7	
3rd quartile	69.6	94.2	
Least poor quartile	85.3	92.9	
	0.0721	0.0131	-0.0591
Concentration index (95% confidence interval)	(0.0347, 0.1100)	(0.0004, 0.0257)	(-0.0981, -0.0201)
***Newborn care practices***			
Breastfeeding within the first hour of birth*			
Overall	74.2	94.0	
Poorest quartile	69.6	92.4	
2nd quartile	69.6	95.1	
3rd quartile	78.9	94.2	
Least poor quartile	79.5	94.2	
	0.0392	0.0013	-0.0379
Concentration index (95% confidence interval)	(0.0054, 0.0729)	(-0.0096, 0.0122)	(-0.0729, -0.0029)
Delay in bathing of at least 6 hours*			
Overall	65.4	81.2	
Poorest quartile	65.6	84.0	
2nd quartile	56.0	86.3	
3rd quartile	66.0	77.2	
Least poor quartile	73.1	77.3	
	0.0153	-0.0252	-0.0405
Concentration index (95% confidence interval)	(-0.0355, 0.0660)	(-0.0465, -0.0038)	(-0.0948, -0.0139)
Skin-to-skin contact between mother and baby immediately after birth**			
Overall	NA	66.1	
Poorest quartile	NA	55.6	
2nd quartile	NA	69.5	
3rd quartile	NA	68.3	
Least poor quartile	NA	71.1	
	NA	0.0492	NA
Concentration index (95% confidence interval)		(0.0189, 0.0796)	

*Observations with missing data excluded from the indicator calculation at baseline. For breastfeeding within the first hour, 17.4% of observations were missing data. For delayed bathing, 9.7% of observations were missing data. **Not measured at baseline.

## Discussion

The CBMNC package in Malawi aimed to contribute to efforts to increase coverage of facility-based care and targeted newborn care practices, and to improve the equity of these services and behaviors. To achieve this aim, SNL and the MOH trained 622 HSAs in the CBMNC package and the intervention reached 38% of women through antenatal home visits and 11% through postnatal home visits. Over the course of the study period, coverage rates significantly increased for facility-based services, newborn care practices, and knowledge. At the same time, Demographic and Health Surveys from 2004 and 2010 demonstrate an increase in use of facility-based maternal and newborn health services across the country [27,43]. Our analysis of baseline and endline household surveys in the study area demonstrates that health equity for knowledge of danger signs and facility delivery, as well as early breastfeeding and delayed bathing, which are promoted during training of facility providers, also improved significantly over the study period. The baseline coverage differences between poorer and wealthier households were relatively small, and accordingly the decreases in inequity were small but significant. Greater improvements in inequities were observed for knowledge indicators. At endline, a greater proportion of women from the poorest quartile reported knowledge of danger signs for pregnancy, delivery, and postpartum mothers than women from the least poor quartile.

Although these results indicate promising improvements for newborn health in Malawi, the extent to which CBMNC contributed to these improvements in coverage and equity are not known. The design of this study would be considered an adequacy design [44], with the lack of a comparison area affecting our ability to attribute coverage and equity changes to the CBMNC program. Program implementation challenges presented additional study limitations. First, the training of HSAs in the pilot area was interrupted for six months due to funding constraints, and the program achieved only moderate coverage of pregnancy home visits and low coverage of postnatal home visits within 3 days of delivery. Additionally, HSAs in Malawi are recruited centrally rather than from the communities in which they serve, and many HSAs were not living at their posts. The HSAs are also expected to spend one or more days per week helping out at health facilities. These factors would likely have reduced their effectiveness at identifying and visiting pregnant women and new mothers. The response of the program to these challenges was to advocate with district and national officials to reinforce residency requirements for HSAs and to encourage communities to provide housing for HSAs in areas where this was a barrier to their residency and to help notify HSAs of pregnant women and newborns in their communities.

At endline, less than 40% of women received a home visit from an HSA during pregnancy. It is not known whether other health communications programs, such as those delivered by clinicians at health facilities or over the radio or text message, influenced the measured outcomes. The decision by the Government of Malawi to ban the use of traditional birth attendants in 2007—a ban which was relaxed in 2010 [45]—likely also affected the utilization of facility-based services.

An additional important component of the program was efforts to improve facility-based care. It should be noted that these quality improvement efforts were limited to government health facilities, and some households, particularly the less poor, may choose other providers, such as the Christian Health Association of Malawi. Nonetheless, there is strong evidence that clients’ perceptions about the quality of care provided at health facilities influence utilization of maternal health services [46-49]. Facility assessments conducted by SNL demonstrated that, although SNL trained and regularly supervised providers, some supply side barriers to quality remained. For example, at one assessment during the study period, less than half of intervention facilities had all four of the following essential newborn commodities available and functioning: injectable gentamycin, thermometer, infant scale, and resuscitation bag and mask [50]. Still, the rates of antenatal care attendance and facility delivery in the endline sample for this study were comparable or higher than those reported by the 2010 Malawi DHS [27].

The results of this study suggest that community-based promotion of key maternal and newborn health behaviors, accompanied by strengthening of facility-based care and counseling, may help reduce inequities in maternal and newborn health. Similar studies in other settings have shown mixed results regarding the ability of community-based programs to improve equity. While evaluations of some community-based programs for maternal and child health report reductions in inequities [51-53], others show that the richest households receive greater benefits from these programs [54,55]. Richer households in the endline survey for this study were more likely to receive a home visit during pregnancy, but were no more likely to have a postnatal home visit or to know of a core group in their community. The observation that the poorest households are not the primary beneficiaries of HSA services is consistent with other assessments of HSA activities in Malawi [56]. The MOH has noted that HSAs posted to the most remote areas are less likely to reside in their catchment areas, and in response to this evaluation and other evidence, is identifying strategies to improve coverage by increasing residency within the hard-to-reach-areas. These results also suggest that the approaches through which community-based programs are implemented likely play an important role in their equity and deserve further attention. For example, the selection criteria for CHWs, their service location, and the presence or absence of pro-poor targeting strategies are all likely to affect equity [6]. The conditions under which community-based programs are successful at reaching the poorest households remains an important research area [17], and evidence suggests that community-based interventions are not automatically guaranteed to improve equity to the extent that policy makers and program implementers may expect.

The implementation context of community-based programs is important when considering program equity results, including the degree of overall wealth inequality in the program setting. Malawi is ranked 70^th^ among nations in terms of wealth inequality [57], and 90% of the population is considered poor by global standards [29]. The differences between households in the poorest categories will be minor compared to other African countries with high wealth inequality, such as Namibia, South Africa, and Botswana [57]. The patterns of wealth inequality also vary by setting, as does the health care system. For example, in Latin America, a greater proportion of the poor population lives in urban areas compared with Africa and Asia [58]. Therefore, local context should guide pro-poor targeting efforts.

As governments in low-income countries increasingly turn towards community health worker programs to improve health outcomes, the appropriate expectations for community-based health workers’ (CBHWs) scope of work and coverage targets should become a focus of policy makers and researchers [59-61]. In Malawi, HSAs, who are traditionally responsible for health education and sanitation activities, are being utilized in task shifting efforts to provide community case management [62], voluntary counseling and testing for HIV[63], and other services. CBHWs in other countries are receiving a similar increase in the scope of their work, which has implications for the impact of individual interventions as well as equity—the workload on CBHWs will likely affect their ability to reach out to the poorest households in the communities they serve.

## Conclusions

Between 2007 and 2011 the study areas is rural Malawi demonstrated a significant improvement in the coverage of knowledge of danger signs and some health behaviors. Some of these general increases reflected small improvements in equitable coverage. The CBMNC package aimed to improve equity by building demand for facility-based services in communities and reducing barriers to counseling for newborn care and maternal and newborn complications. The coverage of home visits by HSAs during pregnancy and the postpartum period was lower than expected, and generally did not significantly favor the poorest households. Strategies for further increasing pro-poor coverage are needed to further reduce inequities in maternal and child health.

## Competing interests

TG, DS, RL, EC, and EZ are employees of Save the Children. FK is employed by the Malawi Ministry of Health. The authors have no other competing interests to declare.

## Authors’ contributions

JC and AB designed the secondary equity analysis of the pilot study data. NB, JC, and RS developed the analysis methods. JC conducted the analysis and wrote the first draft with assistance from NB, TG, and DS. The original pilot study design was supported by TG and FK, and data collection was supported by RL, EC, and EZ. All authors read the manuscript, provided feedback, and approved the final manuscript.

## Pre-publication history

The pre-publication history for this paper can be accessed here:

http://www.biomedcentral.com/1471-2458/13/1052/prepub

## Supplementary Material

Additional file 1: Table S1 Description of Indicators. Click here for file
