# Supplementary material for: Contribution of community-based newborn health promotion to reducing inequities in healthy newborn care practices and knowledge: evidence of improvement from a three-district pilot program in Malawi
Source: BMC Public Health. 2013 Nov 7;13:1052. doi: 10.1186/1471-2458-13-1052 (PMC3833651; doi:10.1186/1471-2458-13-1052)
Supplement: Additional file 1: Table S1 — Description of Indicators. [file 1471-2458-13-1052-S1.docx]

| **Supplemental Table 1. Description of Indicators** | |
| --- | --- |
| **Indicator** | **Description** |
| **Community-based interventions** |  |
| At least one home visit by HSA during pregnancy | Woman reports that an HSA visited her at home at least once during her last pregnancy. |
| Two or more home visits by HSA during pregnancy | Woman reports that an HSA visited her at home at least two times during her last pregnancy. |
| At least one postnatal home visit by HSA within 3 days | Woman reported that the newborn received at least 1 postnatal home visit within three days of birth from an HSA. |
| Two or more postnatal home visits by HSA | Woman reported that the newborn received at 2 postnatal home visits from the HSA, regardless of the time period during which these visits occurred. |
| Aware of a "Core Group" present in community | Woman responds yes to the question: "does your village have a group of men and women (core group) who discuss and disseminate health problems related to pregnancy and child birth?" |
| Received visit from core group member during pregnancy | Woman reports being aware of a core group, and responds yes to the question: "During your pregnancy with [your most recently delivered child], did any members of this group come and visit you to discuss your pregnancy and/or care of the newborn?" |
| **Maternal Knowledge of Danger Signs** |  |
| Knows 3 or more pregnancy danger signs | Unprompted, the woman correctly named 3 or more pregnancy danger signs out of the following list of 12 danger signs: fever; shortness of breath; bleeding; convulsions; weakness; swelling of hands and feet; headache; severe abdominal pain; dizziness; excessive vomiting; blurred visions; less/no fetal movement; ruptured membranes before term. |
| Know 3 or more delivery danger signs | Unprompted, the woman correctly named three or more childbirth danger signs out of the following list of 6 danger signs: heavy bleeding; preterm rupture of membranes; prolonged labor; delay in delivering placenta; asphyxiated baby. |
| Know 3 or more postpartum danger signs | Unprompted, the woman correctly named 3 or more postpartum danger signs out of the following list of 10 danger signs: fever/chills; excessive bleeding; foul smelling discharge; convulsions; abdominal pain; difficulty breathing; urine dribbling; pain in the perineum; feeling weak/faint; swollen or tender breasts. |
| Know 3 or more newborn danger signs | Unprompted, the woman correctly named 3 or more newborn danger signs, out of the following list of 12 danger signs: small/premature baby; jaundice/yellow skin; red swollen eyes or eye discharge; hypothermia/cold temperature; difficulty breathing, fast breathing, or shortness of breath; redness or discharge around the cord; infant is still/rigid; poor sucking or feeding; diarrhea; fever; weakness or little to no movement or crying; convulsions. |
| **Maternal and Newborn Care at Facility** |  |
| At least 1 ANC visit with a skilled provider | The woman reported having attended ANC at least one time and being attended by either a doctor/clinical officer or nurse/midwife. |
| Delivered at a Health Facility | The woman reported delivered at a public or private health facility of the following type: government hospital; community rural hospital; health center; dispensary; health facility operated by the Christian Health Association of Malawi; private hospital; private clinic; private maternity home; other private health facility. |
| **Newborn Care Practices** |  |
| Breastfeeding within the first hour of birth | Woman reported starting to breastfeed the newborn immediately or within 1 hour of birth. |
| Delay in bathing of at least 6 hours | Woman reported that the baby was not bathed during the first 6 hours of after birth. |
| Skin-to-skin contact between mother and baby immediately after birth | Woman responds yes to the question: " Was [your most recently delivered child] placed in skin-to-skin contact with you as soon as s/he was born?" |
